# Life - A glimpse into the phenomenon

**DOI:** 10.6026/973206300221871

**Published:** 2026-03-31

**Authors:** Leszek Konieczny, Irena Roterman, Pawel Spolnik

**Affiliations:** 1 Department of Medical Biochemistry, Jagiellonian University - Medcial College, 31-034 Krakow, Kopernika 7, Poland; 2 Department of Bioinformatics and Telemedicine, Jagiellonian University - Medical College, 30-688 Kraków, Medyczna 7, Poland

**Keywords:** Life, surviving, automatism, self-sustaining

## Abstract

Advancement to knowledge is the possibility to simulate the living system in in silico technique. Therefore, it is of interest to seek an unambiguous interpretation of life as a phenomenon. To overcome the challenges posed by the complexity of biological information, the scope of this study is narrowed down to the cellular level with cells are categorized into two groups: dividing cells - species life, associated with reproduction and non-dividing cells - individual life. This analysis focuses exclusively on individual life, using the erythrocyte as a model for non-dividing cells where the autonomy as a process dedicated to maintaining the stability of the cell's internal environment is treated as the basis. This capacity to sustain internal conditions is recognized as the primary manifestation of life using an automatic response mechanism to environmental changes, governed by negative feedback loops. Furthermore, self-organization, independent recognition of errors and their remediation are identified as key components of autonomous cellular action, the simulation of which is possible using the incorporated simulation tools to model these selected biological processes.

## Background:

Life is a phenomenon that has intrigued mankind for centuries. Initially, attempts to explain it were based on the deliberations of philosophers [1]. In times when science focused its interest on understanding the workings of nature, it was found that the phenomenon of life is distinguished by autonomy of action and traits of independence in living creations that set them apart from their environment; these were considered as a proof that life is an automatic mechanism [2, 3]. Today, there is no universally accepted definition of life. Instead, there are sets of attributes that constitute life. Some of the most prominent modern scholars have spoken on this issue, including Schrödinger, Monod, Crick, or Hawking [4, 5, 6- 7]. In colloquial terms, we often use the term 'life' to denote different kinds of manifestations of social life; an example is 'city life' treated as the collective occupying a limited area of space determines the autonomy of such location and its certain independence from its surroundings, while defining the continuity of duration [8].

We also talk about the birth, life and dying of stars [9, 10]. The definition of autonomy of action also includes artificial creations, such as robots. As science evolved, it became possible to define what is the minimum set of genes essential to sustain basic life function or to form, even by artificial means, minimal cells [11]. Such scientific research enabled theoretical debates about LUCA (last universal common ancestor), the purported common ancestor of all living creations [12]. Therefore, it is of interest to report the treatment of life as automatic activity.

## Materials and Methods:

The processes considered fundamental for the functioning of a living organism are based on the laws of nature arising from the principles of physics and chemistry. The assumption to treat life as an automatic process requires reference to mechanical systems familiar to specialists in technical solution engineering. Combining the knowledge of automaton design proves to necessary in this case. The tools developed here, which are the preliminary form of the proposed model, demonstrate biological processes using simple mechanical principles. The central idea is to consider a system of biological processes as an automaton.

## Results:

To enable a more complete analysis and unambiguous definition of life, science refers to the simplest natural living system such as cells. We clearly consider cells to be living creations. Within this category we distinguish between cells that divide-capable of proliferation - and cells that do not divide, like brain cells, for example, which of course are also alive. The notable representative of cells that do not divide but satisfy the conditions for autonomous action and are capable of ensuring the stability of their internal environment is the erythrocyte. This cell thus satisfies the conditions of independence by ensuring the stability of the internal environment despite the different conditions of the external environmental to which it is exposed while performing its biological function. The erythrocyte is repeatedly walking through in the kidneys and in the lungs during its function's lifetime. It faces extremely varying conditions in those organs successively which results in osmotic stress and oxidative stress alternately [13, 14, 15-16]. The lifespan of nucleated and red blood cells is also determined by nature itself, introducing mechanisms that regulate the duration of functioning and the timing of cellular death [17, 18, 19, 20, 21, 22, 23-24]. In proliferating one, this is a mechanism using telomere shortening, while in the red blood cells, specific protein rearrangements in the cellular membrane determine the ageing and thus the lifespan-of the cells [25]. The mechanisms that ensure the homeostasis in nucleated cells and red blood cells are equipped with their own energy supply. The energy supply in eukaryotic cells is mainly coupled with water synthesis in mitochondria, while the energy resource management in red blood cells is based on glycolysis and the pentose phosphate pathway occurring in the cytoplasm (Figure 1 - see PDF) [24, 25]. In terms of models, the life of those capable of division can be regarded as the life of the species, as the genetic material of cells is duplicated and the flow of information to descendant generations is ensured. In contrast, the life of non-dividing cells, including red blood cells, can be regarded as individual life. Only dividing cells can form structures which condition the process of life. The processes of biological structure formation also exhibit traits of independence and spontaneity. This is expressed in the formation of cell membranes and cytoskeleton, along with protein folding. These are processes of self-organisation, which is the spontaneous formation of structures. It is therefore an expression of independent action.

Structures such as the cell membrane are formed from simple components, which in turn are built of polar and non-polar elements linked together (amphipathic molecules). Introduced into the aqueous environment, they spontaneously form complex membrane systems through the association of these simple components in a self-organisation process. It is possible provided that the non-polar fragments are encased in polar fragments, ensuring the contact of the final product with water (Figure 2 and Figure 3 - see PDF) [25]. The simplest formation of this type is the micelle, while the structure that is arguably important in the spontaneous emergence of life is the liposome [26]. In reality, however, these are time-limited processes generally related to cell division and membrane formation.

The driving force for self-organisation processes comes from the water environment, which, by providing an appropriate external force field, actively directs self-organisation processes by juggling the polar and hydrophobic elements of membrane-building molecules, shaping the three-dimensional structure of proteins using polar and nonpolar amino acid side chains. A tool exists for the assessment of the degree to which the structuring of the hydrophobicity distribution, which is represented to the highest degree by the ordering of micelles [27]. A tool to assess the degree of micelle-like ordering/disordering is available at https://hphob.sano.science/.

This tool determines the structure of a protein using two parameters:

[1] RD, which assesses the extent to which micelle-like ordering has been achieved. This parameter takes values in the range (0-1), where a value of RD=0 indicates full ordering consistent with the hydrophobicity distribution expressed by a 3D Gaussian function- micelle-type arrangement [27].

[2] K, whose positive value is not limited, this parameter indicates the directional contribution of the environment to the folding process. It does not change the spontaneous nature of the process. The effect of the environment only determines the direction of the ordering process of the hydrophobicity distribution appropriate to the local conditions. The higher the value of the K parameter, the stronger the disruption of the polar aqueous environment by the introduction of factors (like the presence of hydrophobic compounds) that modify the matrix to which the folding protein spontaneously adapts its structure.

The product of the server in question is the profile of T, O and M distributions. The T-distribution expresses the distribution of hydrophobicity levels assuming a full micelle-like alignment with maximum hydrophobicity at the centre and zero hydrophobicity at the surface (fit to a 3D Gaussian function). The O-distribution presents the levels of hydrophobicity observed that are the result of hydrophobic inter-residual interaction. The M-distribution is the T-distribution modified by the value of the K parameter expressing the contribution of other non-aqueous environmental factors (the membrane, prefoldin, chaperone and chaperonin). The value of this parameter is given in the legend of the graphs. Identification of residues exhibiting Oi levels congruent with Ti allow the identification of positions (chain segments) locally reproducing the micelle like system ensuring protein solubility in aqueous media. The identification of residues showing significantly different levels of Oi versus Ti allows determination of cavity locations (Oi levels < Ti) and the potential location that is ready to interact with another protein by mediation of hydrophobic interaction (the exposure of hydrophobic residuals on the surface) (Oi > Ti). Such an analysis is represented by an example lysozyme analysis (Figure 4 - see PDF).

[1] Summary of T, O and M profiles for K = 0.4. On the axis, the red dots identify residuals with a deficit of hydrophobicity, while the blue points identify residuals with a local excess of hydrophobicity. The same colours identify the positions of the residuals on the 3D presentation in B.

[2] The 3D structure of the protein in question with highlighted residues showing a local hydrophobicity deficit - ligand or substrate binding cavity (red). Catalytic residues are present among the residues revealing a local deficit. The identification of residues with an Oi < Ti status can provide a method for identifying the location of the biological activity of a protein. The residues showing local excess of hydrophobicity (blue) may be a source of the signal for the surrounding water and consequently, for other molecules.

The status of full conformity of the hydrophobicity distribution to a 3D Gaussian-compatible arrangement only introduces a record of the high solubility of a protein. Specificity, which determines the biological activity of a given protein, is recorded as non-conformity to the expectations of the aquatic environment. The types of non-conformities could theoretically be infinite. If the disorganised form is a method of writing a specific code of biological activity, the spectrum of the encoding possibilities is infinite. In addition, the local record of non-conformity with the expectation of the environment introduces an element of a communication system in the form of imposing on the water molecules a form-compatible ordering/disordering of the water molecules. This can act as a code read by selected molecules present in the environment and their corresponding response to this ordering/disordering of water molecules. The elimination of the residuals highlighted as representing a local non-compliance to the micelle-like system results in an RD = 0.353 and K=0.2. The part of the chain after elimination of the residues indicated as carrying the encoded information remains in the micelle-like system ensuring the solubility of the lysozyme. 3.3. Negative feedback loop at the root of automatism in the process of life - Allosteric structures underlying the automatism in the process of life The ability of receptor structures to form specific connections with signalling elements from other control circuits allows mutual coupling AND expansion of control systems. The proteins showing allosteric forms are proteins with 'decision- making' power. Depending on the allosteric form, the signal output can either initiate or inhibit a particular process. Tracking the effects of changes in the parameters of a negative feedback loop system (limited in size - up to three negative feedback systems) is possible using the software suite at https://nfs.sano.science. The tool that simulates a negative feedback system makes it possible to assess the impact of disturbances of one system on a second system coupled to it. One negative feedback cycle consists of the receptor receiving the signal and the associated effector (the enzyme performing the process). The receptor picks up the concentration of the effector product and, when the encoded level (receptor sensitivity) is exceeded, sends an inhibition signal to the effector. The result is a sinusoidal variation of concentration, which still maintains a certain range of these concentrations (these ranges are known from clinical examinations of the components of body fluids, where the normal (correct) ranges are given). Exceeding these ranges is identified as pathology. The actual mechanism on which the phenomenon of life appears to be based is the process of continuous maintenance of the stability of the internal environment within the cell. This is provided by an automatic process that is based on feedback inhibition (Figure 5 - see PDF) [29, 30-31].

The precondition in nature for the automatic process based on feedback inhibition to emerge was the development of the allosteric mechanism. This mechanism acts like a switch that is operated conditionally; its particular condition of operation is the concentration of components inside the cell. Its particular condition of operation is the concentration of specific cellular components such as substrates and products inside the cell. The operation of this mechanism is ensured by the production of proteins that, when folded, reveal two potential possibilities of stabilisation expressed by different folds with similar energy levels of stability. One of these structural folding forms can bind the substrate and initiate product synthesis. The other form corresponds to the structure of the protein capable of binding the product once its concentration is high enough, resulting in inhibition of synthesis. The concentration of products to which a protein reacts by inhibition depends on the affinity which is evolutionarily determined. This program is the product concentration, controlled by the cell. As a result, the allosteric mechanism enables independent control of the synthesis and concentration of components in the cell and, consequently, the maintenance of the continuity of optimum conditions (Figure 6 - see PDF) [32, 33, 34-35].

Automatism seems to be the very foundation of the phenomenon called life. The emergence of allosteric proteins in nature can therefore be seen as a key step in the emergence of life on Earth. Naturally, there are many automatically controlled processes operating in the cell. They all need to work together. The metabolic products of one feedback inhibition circuit are substrates for other regulatory circuits, doubling as signals which modify the activity of the allosteric proteins in such circuits (Figure 7 - see PDF). These are called allosteric effectors. These interrelations ensure that the cell's internal environment works together and define its optimum conditions. The allosteric receptors represent the power in the form of decision-making regarding other regulation systems. Signal sent by allosteric receptor may initiate or inhibit the process in the cycle coupled with it. Metabolic products serve as signals for normal cell activity and the synthesis of these products is a relatively slow process, the volume of space in which the vital process takes place must be very small. This allows changes in conditions to be timely read as signals. Therefore, the size of individual cells is necessarily very small, independent of the overall size of the organism, whether it is a mouse or an elephant. However the organism, in which automatism is foundational to regulation, takes up a large volume of space compared to the size of a cell. The signalling mechanisms for an organism must therefore be different. In the body, the effectors are specialised cells and their signals are transmitted via neural pathways or hormones. Note that even a very small amount of a hormone in the blood that reaches cells causes a strong effect. This strength of effect stems from a high amplification of the input signal in the cell as it holds amplifiers that work in a cascade [36, 37, 38, 39, 40, 41, 42- 43]. It is therefore a process similar to the operation of a radio, whereby a weak signal reaching the receiver is amplified. As a result of the cellular amplification mechanism, the organism becomes hierarchically superior to the cells (Figure 8 - see PDF).

The tool that simulates a negative feedback system makes it possible to assess the impact of disturbances of one system on a second system coupled to it. One negative feedback cycle consists of the receptor receiving the signal and the associated effector (the enzyme performing the process). The receptor picks up the concentration of the effector product and, when the encoded level (receptor sensitivity) is exceeded, sends an inhibition signal to the effector. The result is a sinusoidal variation of concentration, which still maintains a certain range of these concentrations (these ranges are known from laboratory measurements, where the normal (correct) ranges are given). Exceeding these ranges is identified as pathology. Tracking the effects of changes in the parameters of a negative feedback loop system (limited in size - up to three negative feedback systems) is possible using the software suite at https://nfs.sano.science. The effect of changes in system parameters in product concentration for a single negative feedback is illustrated in (Figure 9 - see PDF). Tracing the consequences of this change to another negative feedback system for the relationship between the two units mediated by the communication addressed to the second-cycle effector is illustrated by the juxtaposition of the two trends in (Figure 10 - see PDF).

The effects of the introduced change representing a disturbance being an increase in the number of effector revolutions when the communication is mediated by the effector affects the second system in the form of an increase in the frequency in the second system (Figure 10 - see PDF). In contrast, when communication is mediated through the receptor, changes in receptor parameters in both systems are illustrated in (Figure 11 - see PDF). The examples cited illustrate the behaviour of abstract negative feedback systems to a very limited extent (there are only 3 negative feedbacks).

It is assumed that the expansion of a system of such interdependent units could eventually enable any expanded system to function. The intentional disturbance of a single negative feedback system can reveal the propagation of the effects of this disturbance and pinpoint the (sometimes distant) location in the system where an already introduced change manifests itself on an observable outputs. However, not all processes follow the pattern proposed above. There is, however, an exception. There are processes in nature where a cell uses an amplification mechanism independently. Here, an example is mainly the immune system cells, distributed throughout the organism. Similarly, during hemostasis and clot formation, platelets also act in an autocrine manner. An immune cell can act on its own when it has local contact with an antigen. This works by the immune cell sending out a hormone-like product that binds with a specific receptor along the hormonal signal pathway; the receptor features a signal amplifier. This allows the amplified signal to reach the cellular 'clockwork' responsible for cellular function. It is how a cell can amplify its activity on its own. The cell simply commands itself to activate. We call this process autocrine signalling. It is a form of self-regulation [42, 43]. The autonomy of action associated with the process of living is also expressed through certain biological solutions such as the repair processes of error identification and error rectification mechanism. This is yet another expression of the independence of nature's creations [44, 45]. A remarkable feature of life is its ability to survive in harsh conditions; this includes the viability of seeds that is sustained for years [45, 46]. Simplification of mechanisms as a route to reliability a well-known example of information volume reduction in nature by replacing qualitative traits with a large quantity is the production of multiple seeds by plants. This solution ensures certainty by using probability [47]. The objective can be certainly achieved by carefully recording the conditions for its emergence. This entry is defined by biology as specificity, including, for example, the specificity of substrate selection by the enzyme, but also the interaction with a messenger (hormone) addressed to a selective target. This solution requires accurate recording of all recognition elements in order to increase the probability value, p, of reading the message correctly. This is achieved by appropriately specific structures with coded possibilities for specific selective interaction. Another solution to achieve the objective without recording the recognition code is to mass-produce the distributed information in the hope that at least one of the messages will be read correctly. This is expressed in the repeated repetition of a specific act without a defined purpose. This multiplicity, expressed by the k value, makes it possible to determine at what number of repetitions the objective will be achieved. A classic example of this is the functioning of the immune system, where the target (antigen) is unpredictable and indeterminate. Therefore, the mass synthesis of antibodies with a variable target identification element ensures the recognition of the antigen and the initiation of an immune response. Another example is the mass production of seeds on the assumption that one of them will fall on a site where a new progeny plant can grow. A tool that illustrates the relationship between the recording of specific information (p) and the need to repeat a process (k) is a software suite available at https://ip.sano.science.

The analysis carried out with this tool aims to find the optimal set of p and k to achieve the objective with the minimum energy input. The degree of coding required to record with specificity (p) can be lower if supported by a sufficient number of iterations (k) (Figure 12 - see PDF).

## Discussion:

It seems to be connected to the simplicity of mechanisms employed by nature and thereby their reliability. The evolutionary mechanisms of adaptation of organisms to environmental conditions achieved by random mutations and subsequent trait selection are well known. However, there is a strategy found at a cellular and molecular level, aimed at bypassing the need for information input. Reliability is generally related to the simplicity of a mechanism. An example of a solution for the reliability of mechanisms is the employment of self-organisation in the formation of biological structures such as cellular membranes and protein folding. Self-organisation is driven by entropic mechanisms and does not require a continuous input of information to control the process. An example of another mechanism is the relatively simple principle of intracellular regulation that enables using metabolic products directly as signals, which in effect essentially simplifies the need for information input. It is possible thanks to the small volume of space in which those controlled processes take place. This mechanism is based on allosteric proteins (enzymes). Self-organisation and simple regulatory mechanisms not only reduce the need for continuous information input but also increase the reliability of cellular processes. This simplicity allows organisms to function efficiently even under conditions that are variable or unpredictable. A striking illustration of this principle is the ability of certain organisms to colonize highly unfavorable environments (Figure 13 - see PDF) [46, 47], where the robustness of simple, self-organising mechanisms ensures survival despite extreme conditions. The general interpretation of homeostatis requires the basis in form of negative feedback loops network [48, 49, 50, 51, 52, 53, 54, 55, 56, 57, 58, 59, 60, 61, 62, 63-64]. This is the only system ensuring mutual control of all processes in organism. The accessibility of tosls allowing the experiments in silico makes possible the simulation of experiments in silico [27, 65 and 66].

## Conclusion:

Science discovers and explains the mechanisms and processes to understand the essence of life; still, life remains an admirable and extraordinary phenomenon. Although many questions remain unanswered or answers to them are sought in the field of philosophy, science offers an interpretation of the foundations of the process of life using the universal assumptions of physics and chemistry; nevertheless, it emphasizes the extraordinary nature of life, which we face with awe. The stability expressed as homeostasis for organism which is the open system can be ensured solely by network of negative feedback loops.

## Data accessibility:

Three tools available in open access systems allow the results described to be obtained in relation to:

[1] Assessing the type of ordering of hydrophobicity distribution in a protein body: https://hphob.sano.science/

[2] Relationship of the degree of information encoding to the number of times a process is repeated in order to achieve a specific objective: https://ip.sano.science.

[3] Simulating the operation of the negative feedback loop system: https://nfs.sano.science.

## Author contributions:

All Authors of equal contribution.

## Informed consent statement:

Not applicable.

## Data availability statement:

Not applicable. The tools allowing simulation of discussed issues are available in open access. Addresses are given.

## Funding statement:

No funding received.

## References

[1] Long AA (1966). The Classical Quarterly..

[2] https://plato.stanford.edu/.

[3] https://www.biodiversitylibrary.org/.

[4] https://www.abebooks.com/.

[5] https://ia800107.us.archive.org/25/items/brief-answers-to-the-big-questions/Brief%20Answers%20to%20the%20Big%20Questions.pdf.

[6] https://dokumen.pub/life-itself-9780671255626-0671255622.html.

[7] Crick F (1981). Life Itself: Its Origin and Nature..

[8] Nugent A, Nugent P (2025). Nature..

[9] Braeckman BP (2002). Aging Cell..

[10] Vitas M, Dobovisek A (2019). Orig Life Evol Biosph..

[11] Glass JI (2017). Cold Spring Harb Perspect Biol..

[12] Woese CR (1990). Proc Natl Acad Sci USA..

[13] Föller M (2008). IUBMB Life..

[14] Hosey MM, Tao M (1976). Biochemistry..

[15] Jürgens D (2002). Cell Biol Int..

[16] Lang KS (2005). Cell Physiol Biochem..

[17] Nandakumar J, Cech TR (2013). Nat Rev Mol Cell Biol..

[18] Giardini MA (2014). Prog Mol Biol Transl Sci..

[19] Liu J (2019). Cells..

[20] Zhu Y (2019). Biogerontology..

[21] Lu W (2013). Exp Cell Res..

[22] Rhodes D (2002). EMBO Rep..

[23] Aguado J (2020). Ageing Res Rev..

[24] Konieczny L (2023). Functional Strategies of Living Organism..

[25] Schiffmann Y (1997). Biochem Soc Trans..

[26] Akbarzadeh A (2013). Nanoscale Research Letters..

[27] Roterman I, Konieczny L (2023). Entropy..

[28] Herning T (1992). Biochemistry..

[29] Konieczny L (2023). Functional Strategies of Living Organism..

[30] Gao H (2022). Rev Endocr Metab Disord..

[31] Basu S (2022). Mol Cell Biol..

[32] Monod J (1965). J Mol Biol..

[33] Imelio JA (2021). Biophys Rev..

[34] Schamel WW (2017). J. Immunol..

[35] Konieczny L (2023). Functional Strategies of Living Organism..

[36] Hiller-Sturmhöfel S, Bartke A (1998). Health Res World..

[37] Yanagi S (2018). Cell Metab..

[38] Akbarzadeh A (2013). Nanoscale Res Letters..

[39] Glass DS, Alon U (2018). Science..

[40] Hutfilz C (2022). Front Physiol..

[41] Schaum N (2020). Nature..

[42] Papin JA (2004). Trends Biochem Sci..

[43] Pandit NK (2007). Introduction to The Pharmaceutical Sciences..

[44] Bafico A (2004). Cancer Cell..

[45] Hanawalt PC, Spivak G (2008). Nat Rev Mol Cell Biol..

[46] Chatterjee N, Walker GC (2017). Environ Mol Mutagen..

[47] Konieczny L (2023). Systems Biology..

[48] Tetz VV, Tetz GV (2020). Biomol Concepts..

[49] Cleland CE, Chyba CF (2002). Orig Life Evol Biosph..

[50] Benner SA (2010). Astrobiology..

[51] Chodasewicz K (2014). Theory Biosci..

[52] Dix DE (2002). Yale J Biol Med..

[53] Tirard S (2010). Astrobiology..

[54] Mix LJ (2015). Astrobiology..

[55] Luisi PL (1998). Orig Life Evol Biosph..

[56] Jeller H (2003). Braz J Biol..

[57] Kangueane P (2001). Hum Immunol..

[58] Sakharkar MK, Kangueane P (2004). BMC Bioinformatics..

[59] Mix LJ (2026). Astrobiology..

[60] Stepney S (2025). Philos Trans R Soc Lond B Biol Sci..

[61] Abel DL (2026). Journal of Bioinformatics and Systems Biology..

[62] Abel DL (2025). Biosystems..

[63] Abel DL (2024). Archives of Microbiology and Immunology..

[64] Abel DL (2011). Life (Basel)..

[65] https://www.ceneo.pl/.

[66] https://shop.elsevier.com/.

